# A multimodal intervention to optimise antimicrobial use in residential aged care facilities (ENGAGEMENT): protocol for a stepped-wedge cluster randomised trial

**DOI:** 10.1186/s13063-022-06323-8

**Published:** 2022-05-21

**Authors:** Nazanin Falconer, David L. Paterson, Nancye Peel, Alyssa Welch, Christopher Freeman, Ellen Burkett, Ruth Hubbard, Tracy Comans, Leila Shafiee Hanjani, Elaine Pascoe, Carmel Hawley, Leonard Gray

**Affiliations:** 1 UQ Centre for Health Services Research, Faculty of Medicine, The University of Queensland, The University of Queensland, Brisbane, QLD 4102 Australia; 2 Department of Pharmacy, Princess Alexandra Hospital, Metro South Health, Brisbane, QLD 4102 Australia; 3 School of Pharmacy, Faculty of Health and Behavioural Sciences, The University of Queensland, The University of Queensland, Brisbane, QLD 4102 Australia; 4 UQ Centre for Clinical Research (UQCCR), Faculty of Medicine, The University of Queensland, Royal Brisbane and Women’s Hospital Campus, Brisbane, Australia; 5 Royal Brisbane and Women’s Hospital, Metro North Health, Butterfield Street, Herston, Brisbane, QLD 4029 Australia; 6 Department of Emergency Medicine, Princess Alexandra Hospital, Woolloongabba, Brisbane, QLD 4102 Australia; 7 Princess Alexandra Hospital Southside Clinical Unit, Faculty of Medicine, The University of Queensland, Woolloongabba, Brisbane, QLD 4102 Australia

## Abstract

**Background:**

Inappropriate antibiotic use can cause harm and promote antimicrobial resistance, which has been declared a major health challenge by the World Health Organization. In Australian residential aged care facilities (RACFs), the most common indications for antibiotic prescribing are for infections of the urinary tract, respiratory tract and skin and soft tissue. Studies indicate that a high proportion of these prescriptions are non-compliant with best prescribing guidelines. To date, a variety of interventions have been reported to address inappropriate prescribing and overuse of antibiotics but with mixed outcomes. This study aims to identify the impact of a set of sustainable, multimodal interventions in residential aged care targeting three common infection types.

**Methods:**

This protocol details a 20-month stepped-wedge cluster-randomised trial conducted across 18 RACFs (as 18 clusters). A multimodal multi-disciplinary set of interventions, the ‘AMS ENGAGEMENT bundle’, will be tailored to meet the identified needs of participating RACFs. The key elements of the intervention bundle include education for nurses and general practitioners, telehealth support and formation of an antimicrobial stewardship team in each facility. Prior to the randomised sequential introduction of the intervention, each site will act as its own control in relation to usual care processes for antibiotic use and stewardship.

The primary outcome for this study will be antibiotic consumption measured using defined daily doses (DDDs). Cluster-level rates will be calculated using total occupied bed numbers within each RACF during the observation period as the denominator. Results will be expressed as rates per 1000 occupied bed days. An economic analysis will be conducted to compare the costs associated with the intervention to that of usual care. A comprehensive process evaluation will be conducted using the REAIM Framework, to enable learnings from the trial to inform sustainable improvements in this field.

**Discussion:**

A structured AMS model of care, incorporating targeted interventions to optimise antimicrobial use in the RACF setting, is urgently needed and will be delivered by our trial. The trial will aim to empower clinicians, residents and families by providing a robust AMS programme to improve antibiotic-related health outcomes.

**Trial registration:**

US National Library of Medicine Clinical Trials.gov (NCT04705259). Prospectively registered in 12th of January 2021.

**Supplementary Information:**

The online version contains supplementary material available at 10.1186/s13063-022-06323-8.

## Background

The World Health Organization (WHO) has declared antimicrobial resistance (AMR) to be a world-wide health crisis [1], with a global rise in antibiotic resistant organisms, resulting in significant increase in healthcare utilisation, morbidity and mortality [2]. In 2017, the WHO published its first ever list of antibiotic priority pathogens—12 groups of bacteria that pose the greatest threat to human health [1]. This burden of AMR is particularly high among elderly residents at long-term care facilities [3–5].

National point prevalence surveys have consistently demonstrated room for improvement in prescribing practices with approximately 10% of residents in Australian residential aged care facilities (RACFs)—long-term care facilities providing 24-h nursing services—using antibiotics at any given time [6, 7]. Data from the Pharmaceutical Benefit Scheme suggests that approximately 70% of RACF residents receive at least one course of antibiotics annually. Internationally, similar figures are reported with between 45 and 79% of residents from the USA, Canada and the UK receiving one or more antibiotics over a 12-month period [8, 9].

This is supported by a recent Australian study by Sluggett et al. that demonstrated an increase in both the prevalence and total consumption of antibiotics in RACFs over a 10-year period [10]. Studies indicate that a high proportion of these prescriptions (25-75%) are non-adherent with best prescribing guidelines and that treatment choice and duration are also frequently inappropriate [6].

There is a direct correlation between overuse of antibiotics and AMR [11]. RACFs are at particularly high risk of AMR as they provide long-term care to individuals who are often frail, with multimorbidity and live in close proximity, making them more prone to infections and subsequent antibiotic use. Furthermore, RACFs operate in a climate of tight resourcing and in many facilities there are no onsite doctors. Out of hours, medical advice is provided by practitioners who do not necessarily know the resident and may not have access to complete medical records. A large retrospective Australian study of antibiotic use in Australian RACFs found greater prevalence of out of hours medical practitioner services associated with greater antibiotic use [12].

RACFs have been identified as complex adaptive systems, with multiple influences on antimicrobial prescribing, including clinical staff, factors related to the facility and the resident and family wishes [13], all of which may play a role in inappropriate prescribing and the development of resistant organisms [14]. This is problematic given that RACF residents change care settings frequently, and resistant organisms may be transferred between the facility and acute care, increasing transmission of AMR [14].

A structured Antimicrobial Stewardship (AMS) model, incorporating multimodal interventions targeted at optimising antimicrobial use and resident outcomes in the RACF setting is urgently needed. AMS has been defined as “a systematic and coordinated approach to optimising antimicrobial use with the goals of improving patient outcomes, ensuring cost effective therapy and reducing adverse consequences of antimicrobial use, including AMR” [15]. Whilst in the Australian healthcare system AMS programmes have evolved significantly in acute care, and are now mandatory for hospital accreditation purposes, stewardship programmes in long-term care facilities are still evolving [16]. Recent Aged Care Quality standards require that data is used to monitor infections and resolution as part of the effectiveness of infection prevention and control programmes [17]. Facilities need to be able to provide evidence-based surveillance reports, with quality and risk interpretation of data [17].

### Key infection types and interventions to improve antibiotic use

The strongest evidence for inappropriate prescribing in the RACF setting relates to suspected urinary tract infections (UTIs), which account for up to 60% of antibiotic courses. Asymptomatic bacteriuria (ASB) (vs. symptomatic UTI) becomes more frequent with advancing age [18]. Antibiotic treatment for ASB has no benefits for the individual and does not reduce mortality, nor the incidence of symptomatic UTI [19]. Studies show that nearly half of antibiotic prescribing in RACFs is for ASB, or based on urine samples collected in residents whose symptoms are unlikely to be UTI related [19, 20].

Antibiotic consumption for other common conditions has also been identified as high and potentially inappropriate. These include respiratory tract infections (RTIs) and antibiotic prescribing in skin and soft tissue conditions. Together with UTIs these conditions account for approximately 90% of all infections (Table 1) [24]. Inappropriate treatment significantly increases the risk of antibiotic-related harm, including *Clostridium difficile* (*C. difficile*) infections and AMR. Ideally, interventions should aim to target all three areas to optimise impact. This is in line with the antibiotics on the WHO Watch list (i.e. those with high resistance potential), which should be targeted by AMS programmes [25].

**Table 1:** Common conditions associated with sub-optimal antibiotic prescribing in RACFs

Conditions	Sub-optimal prescribing examples
Urinary tract infections	E.g. inappropriate prescribing in asymptomatic bacteriuria. Improving diagnosis and treatment of UTIs [5, 18, 21, 22] Inappropriate and widespread use of long-term prophylactic antibiotics for prevention of UTIs
Respiratory infections	E.g. antibiotic use in RTI, including bronchitis. RTIs are usually due to viruses and antibiotic use is inappropriate—there is a need for education and improving diagnosis [22, 23].
Skin and soft tissue	E.g. inappropriate treatment of conditions frequently confused with cellulitis such as venous stasis; inappropriate treatment of ulcer or wound bacterial colonisation [22]. Inappropriate use of topical agents [7]. There is a need for further education and improving diagnosis.

*UTI* urinary tract infection, *RTI* respiratory tract infection

Several interventions have shown positive results for improving antibiotic prescribing practices in the aged care setting [26–30]. Stewardship programmes incorporating educational elements have resulted in improved prescribing and reductions in unnecessary urine testing [26–29]. A systematic review by Davey et al. identified that dissemination of educational resources and audit feedback can assist with better prescribing of antimicrobials in the hospital setting [30]. Other interventions include implementation of site-specific guidelines, provision of telehealth services to facilitate timely advice from expert infectious disease clinicians [31] and use of decision support tools to guide prescribing [32]. A recently published systematic review highlighted studies that use several interventions have better outcomes than those using education only strategies [33]. Recently a multi-centre study by Nace et al. [18] reported a significant reduction of 17% in antibiotic use for asymptomatic bacteriuria through the delivery of a quality improvement intervention across 25 facilities in the USA. The study did not find any adverse events related to the intervention, with no differences in all-cause hospitalisations or resident mortality between the intervention and control groups [18].

A systematic review which specifically evaluated the impact of AMS on resident outcomes and health care utilisation also found interventions to be safe [34]. In this study, eight of 14 eligible studies reported a reduction in antibiotic prescribing [34]. Another review identified 19 studies that evaluated interventions to optimise antimicrobial use in the aged care setting [35]. Studies targeted either UTIs, RTIs or both. Only two studies demonstrated a significant decrease in antibiotic consumption, and no safety concerns were reported (e.g. change in hospitalisations or resident mortality) [35]. Most studies were conducted in the US long-term care facilities (*n*= 14), three in Europe and only one study in the Australian healthcare setting, which evaluated an educational intervention aimed at nursing staff. Studies were deemed to be at high risk of bias due to their design (i.e. not a randomised controlled study (RCT)) [35].

Whilst interventions have shown improvements in prescribing of antimicrobials, there are no studies to date which have evaluated the impact on antibiotic consumption (measured using defined daily doses [DDD]), resident impact (*C. difficile*, AMR and hospitalisations) and cost-effectiveness using a comprehensive set of multimodal interventions in a tailored bundle, as part of a stewardship programmes in the Australian RACF setting. Due to the variation in clinical governance, sociodemographic environments and the approach to medical diagnosis and treatment (e.g. in Australia physicians are generally not located onsite), a robust multicentre study in the Australian RACF context is needed. Furthermore, to date studies have lacked a rigorous study design with a high risk of bias. A study that delivers interventions using a stepped-wedge RCT design, whereby each facility can receive the intervention and act as its own control, could address previous deficiencies. Therefore, our research aims to identify the impact of a set of sustainable, multi-modal interventions, targeting three common areas of antibiotic prescribing using a stepped-wedge RCT design. It is our intention that the learnings from this research translate to sustainable improvements in resident outcomes at RACFs across Australia.

### Aims

1. To determine if the delivery of a bundle of multimodal interventions reduces antibiotic use in RACFs as measured by DDDs.
2. To determine if this bundle has an impact on resident outcomes, including antibiotic-related harm, mortality and hospital admissions.
3. To determine if this bundle is cost effective.

### Study hypothesis

Compared with routine care, implementation of a multimodal intervention bundle (AMS ENGAGEMENT) will be associated with a 20% reduction in DDD post trial intervention period.

The *null hypothesis* for this trial is there will be no change in DDD of antibiotics because of the trial intervention.

## Methods

This protocol was developed in accordance with the Standard Protocol Items: Recommendations for Interventional Trials (SPIRIT) Statement [36]. The completed SPIRIT Checklist is included in the Additional file 1. Reference was also made to CONSORT statement extension for cluster randomised trials and the recommended modifications for stepped-wedge designs [37].

### Design and setting

**Fig. 1:**
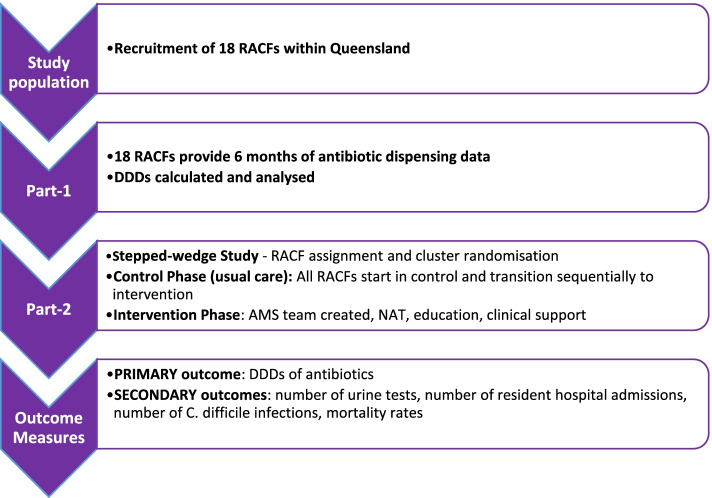
The trial schema outlining the development and stepped-wedge trial (intervention and control). AMS, antimicrobial stewardship; DDD, defined daily doses; RACF, residential aged care facility; NAT, Needs Assessment Toolkit

The study will be conducted in two parts, as summarised in Fig. 1:

- Part 1: Trial development. RACF recruitment and refinement of the AMS ENGAGEMENT intervention bundle
- Part 2: Stepped-wedge trial

### Part 1: Trial development

#### Recruitment of RACFs

Eighteen RACFs will be recruited from South East Queensland, Australia, for this trial. Aged care service providers with facilities of 50 or more residents will be directly approached and invited by the research team through local pharmacy providers Service providers and facilities interested in participating will receive a letter of invitation to participate that details the participation requirements and the financial and in-kind support provided by the research team. Those that respond will also be invited to a meeting with members of the trial management committee to answer queries related to their participation in the trial.

#### Data collection

De-identified data will be collected from 18 RACFs for the same 6-month period (July to December 2020) on the following variables: antibiotics usage from pharmacy dispensing data, resident demographics from the RACF records and hospitalisations and mortality from hospital discharge summaries. This data will be used to calculate DDDs to form a reference base for safety monitoring.

### Part 2: Stepped-wedge trial

#### Study population

Residents of 18 Queensland RACFs with at least 50 residents at their facility.

#### Inclusion criteria

Registered RACFs within Queensland, Australia, with at least 50 residents; able to provide the monthly de-identified reports necessary to conduct the study.

#### Exclusion criteria

RACFs with fewer than 50 residents; RACFs unable to provide monthly de-identified study reports.

#### Randomisation of RACFs

The 18 RACFs will be randomly assigned to an intervention starting time in a stepped-wedge RCT. The trial will be conducted across a 20-month observation period. The first month will be a control period for all sites; one site will transition from control to intervention each month for 18 months based on their randomisation; the final month will be an intervention period for all sites.

#### Control period (1–18 months)

Whilst in the control period the RACF will deliver usual care, which involves the facility’s current processes in relation to antibiotic use and the stewardship programmes.

#### Transition period (1 month)

This period will be the time when the intervention will be established by engagement of facility staff, delivery of educational materials to registered nurses and GPs and implementation of protocols and digital resources from the AMS ENGAGEMENT bundle.

#### Intervention period (1–18 months)

**Fig. 2:**
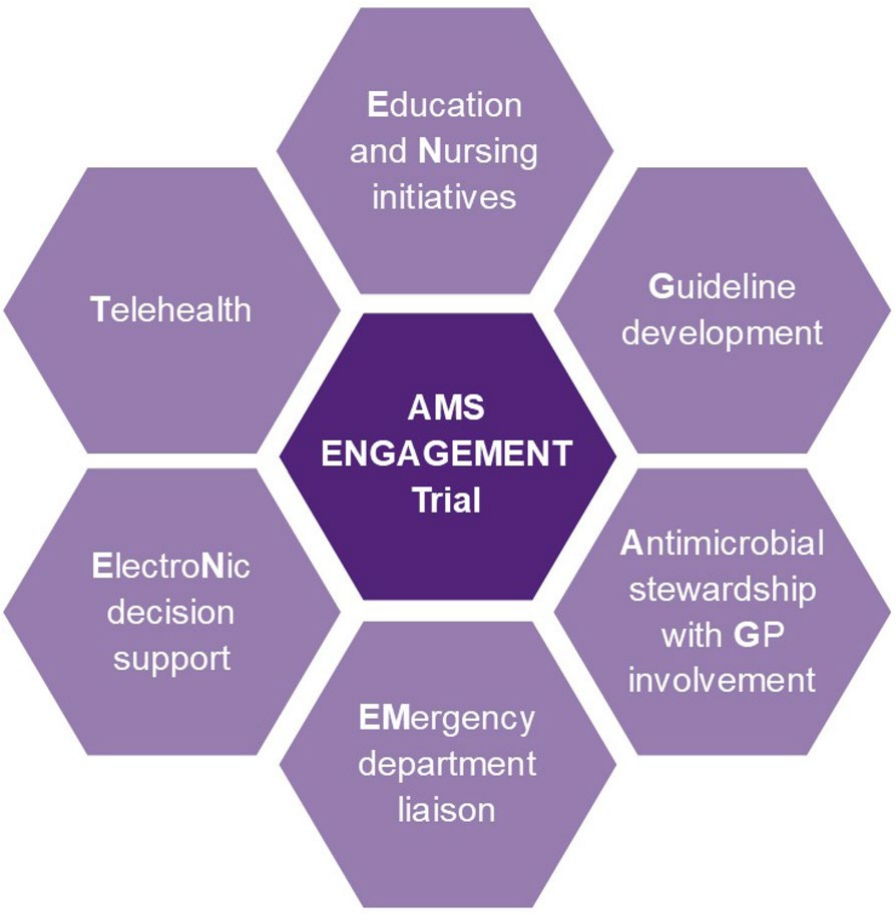
The AMS ENGAGEMENT bundle intervention components

Whilst the RACF is in the intervention period it will receive a set of multimodal and multidisciplinary services, the ‘AMS ENGAGEMENT’ bundle, summarised in Fig. 2 and described in Table 2. The intervention will be delivered at the level of the RACF.

**Table 2:** Key elements of the RACF AMS ENGAGEMENT bundle

Intervention components	Activities	Delivery and phase
Education and engagement of (a) GPs and pharmacists (b) Nurses (c) Residents and their families	Develop information packages on (a) Antibiotic choice and duration (b) Indications for specimen collection, notification of infection-related symptoms to GPs and microbiological testing as clinically indicated (c) Hazards of inappropriate use for RACF residents and families	A 4-week course for RACF nurses with a primary focus on treatment of suspected UTIs. Brochures and posters for residents and families. Academic detailing with GPs. **Induction** to be implemented during the transition period from control to intervention (online-only delivery possible if required) **Maintenance** materials for refresher training and/or new employees available during intervention
Nursing initiatives to enable seamless delivery of the trial	Establish a dedicated telehealth portal (using messaging and telephone communication) to enable RACF nurses to communicate with a researcher team member (on an as-needs basis) with regard to any aspect of the trial.	Phone, email and BlackBoard Web Platform®—using online discussion forums to communicate with an expert member of the research team. **Induction and maintenance**
Guideline development specific to antibiotic use in RACF residents	Evidence-based resident-specific clinical pathways and antimicrobial guidelines will be collaboratively developed with input from emergency clinicians, infectious diseases/microbiologists, geriatricians and general practitioners	State guidelines will be tailored to RACFs based on knowledge of local practices, reviewed annually. **Induction and maintenance**
Antimicrobial stewardship team creation in RACFs	Establish an AMS team at each facility. The composition of this team will include the clinical nursing director of the RACF, infection control nurse, a key GP working in the RACF, a pharmacist from the pharmacy providing quality use of medicine services to the RACF and an antimicrobial steward.	Oversee the stewardship process, review of the antibiotic guidelines for the RACF, review of the suite of interventions which are part of package and review of outcomes. Members of research team will participate in the first two AMS meetings to build capacity. **Induction and maintenance**
Emergency department liaison and promotion of state-wide clinical pathways to ensure consistency of practice across the care continuum	Establishment of an ED liaison to ensure continuity of AMS practices across health care settings	RACF staff to communicate with an ED liaison via phone and /or a letter informing them of the resident’s participation in the trial and key goals of AMS ENGAGEMENT **Induction and maintenance**
Electronic decision support to guide RACF urine testing and GP antibiotic prescribing	Access to mobile technology that provides decision support to underpin antibiotic use among RACF residents.	Access to QH Management of Acute Care Needs of RACF residents Guidelines App to help with diagnosis and prescribing and decision-making. **Induction and maintenance**
Telehealth support for key intervention components	Quarterly webinars with an expert panel on key issues related to AMS and/or practice changes in RACFs	Telehealth to support case discussions, and for education and training. AMS team and expert panel from research team to act as a panel with three monthly teaching sessions and case-based discussion open to all RACF-registered nurses and GPs in the intervention period **Induction and maintenance**

*AMS* antimicrobial stewardship, *GP* general practitioner, *QH* Queensland Health, *RACFs* residential aged care facilities, *UTI* urinary tract infection

#### AMS ENGAGEMENT Bundle Needs Assessment Toolkit

An AMS Needs Assessment Toolkit (NAT) will be developed and refined based on consultation with RACF clinical staff, reviews of the literature and published resources. Existing materials will inform its design, including the Clinical Excellence Commission’s Antimicrobial Stewardship Progress and Planning Tool (for acute care in Australian hospitals but modified for use in the aged care setting) [38] and the Centres for Disease Control and Prevention: “Core Elements of Antibiotic Stewardship for Residential aged care facilities” [39]. The NAT will assess the following points:

- Whether the RACF has the necessary resources for AMS (i.e. staff and methods) to establish clinical governance (current prescriber/General Practitioner (GP) involvement in the stewardship programmes). If an AMS programme exists, explore how the trial can enrich and support this service.
- Whether suitable institutional policies and procedures exist for antibiotic prescribing and administration, in particular with regard to treating suspected UTIs.
- What UTI management practices take place at the facility (e.g. symptom identification, diagnostic work-up, diagnosis, treatment, administration of treatment, review, communication).
- How antibiotic consumption and antibiotics-related adverse events and resistance patterns are identified, tracked and reported.

Each RACF will use the information collected from the NAT to determine key areas for focus and collaboratively develop an action plan prior to implementation, e.g. availability and use of an Imprest Medication System, GP and nurse knowledge on national and local antibiotic guidelines, processes related to urine testing (e.g. use of dipsticks), staff and family member awareness and education on AMR and its prevention. RACFs will also determine the best approach for access to specialist medical advice, monitoring and methods for reporting of AMS metrics.

The NAT will be emailed to the site coordinator ahead of the first AMS workshop and the responses will inform the discussions in the first AMS workshop for each facility. The findings of the NAT will help with refinement and implementation of the AMS ENGAGEMENT intervention bundle that each facility implements during the stepped-wedge trial.

Three months prior to a RACF’s randomised intervention start date, the research team will communicate with the facility to start the process of establishing an AMS clinical governance team (and if one already exists, they will receive support and mentoring from the research group and their resources will be enriched using trial material). This team will be assembled to discuss the NAT in an initial AMS workshop and identify how best to operationalise the interventions within their facility. Based on the NAT results, appropriate interventions will be selected from the AMS ENGAGEMENT bundle at each facility (Fig. 2).

Given that a single intervention is unlikely to be effective in stewardship programmes, nor in care improvement processes at RACFs [40], this trial will deliver a set of multimodal, multidisciplinary interventions to optimise antibiotic use in RACFs. This RACF *AMS ENGAGEMENT* bundle will comprise the following key interventions, which will be informed by the NAT and individual requirements of each facility (Table 2):

- *E*ducation and engagement of prescribers, nurses, pharmacists and residents and family members
- *N*ursing initiatives to improve UTI diagnosis and reduce inappropriate urine testing
- *G*uideline development specific to antibiotic use in RACF residents
- *A*ntimicrobial stewardship team creation in RACF with *G*P involvement
- *EM*ergency department liaison and use of clinical pathways to ensure consistency of practice across the care continuum
- *E*lectro*N*ic decision support to guide RACF urine testing and GP antibiotic prescribing
- *T*elehealth support for key intervention components

The study has been designed to allow the remote delivery of all intervention components (i.e. through use of print materials, videoconferencing, webinars, telephone support and electronic bulletins).

**Fig. 3:**
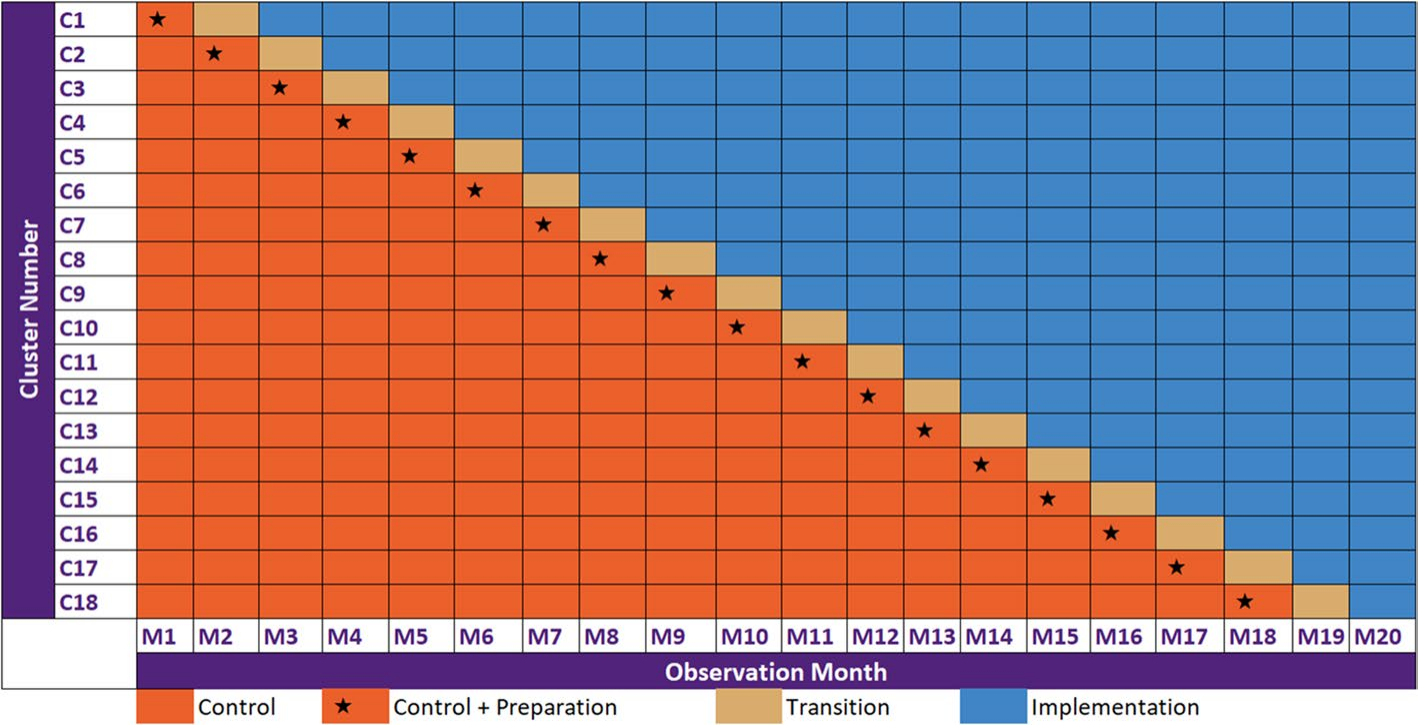
Schematic representation of the stepped-wedge AMS ENGAGEMENT study

The Trial will involve the implementation of an AMS ENGAGEMENT bundle into 18 RACFs using a stepped-wedge-RCT design (Fig. 3).

### Outcome measures

#### Primary outcome measure

The primary outcome measure for this trial is antibiotic use as measured by DDDs of antibiotics per 1000 resident bed days.

#### Secondary outcome measures

- Number of urine samples collected per 1000 resident bed days between the control and intervention periods
- Percent susceptibility of Enterobacteriaceae to ceftriaxone, ciprofloxacin, cephalexin and amoxicillin-clavulanate
- All-cause year-on-year mortality rates of RACF residents between the control and intervention periods (per 1000 resident bed days and median rate across facilities)
- Number of RACF residents admitted to hospital during the control vs intervention periods (per 1000 resident bed days and median rate across facilities)
- Number of cases of *C*. *difficile-*associated diarrhoea between the control and intervention periods (per 1000 resident bed days and median rate across facilities)
- The net cost of the intervention.

#### Exploratory outcome measures

- Barriers and enablers to improving AMS practices in RACFs.

This study will also report outcomes in relation to most of the recently published Core Outcome Set for antimicrobial stewardship [41]. Antibiotic dispensing and resident data reported by RACFs will enable calculation of the total number of antimicrobial courses prescribed that are actually dispensed (incidence of antimicrobial use) per 1000 resident bed days; and days of therapy per 1000 resident bed days (rate of antimicrobial therapy). Infection-related mortality will also be reported (using before and after antibiograms). Appropriateness of antimicrobial prescribing will not be reviewed. No restrictions will be imposed on facilities for undertaking other education or activities related to medication prescribing and optimisation outside of the ENGAGEMENT bundle (e.g. routine facility pharmacist in-service sessions).

### Sample size

Sample size was calculated based on the outcome of antibiotic consumption as measured by DDDs. For a trial to run over 20 months with a mean cluster size of 50 residents and a control event rate of 120 DDDs per 1000 resident days [22, 42], 18 RACFs will provide 82% power to detect a 20% reduction in DDDs, which has been demonstrated as feasible by other studies [28, 43] (although these studies have utilised other metrics to quantify antibiotic consumption). The power calculation is based on a conservative intra class correlation coefficient (ICC) of 0.012 and coefficient of variation of 0.3 [44].

### Statistical analysis

The primary outcome is DDDs of antibiotics per 1000 occupied bed days. DDDs will be measured at the patient level monthly and analysed using a generalised linear mixed model for count data. The model will include fixed effects for intervention (AMS ENGAGEMENT vs control) and calendar time (randomised crossover times) and two random effects, one for variation among RACFs and a second to accommodate repeated measures over time on participants. Analysis will be by the intention-to-treat-schedule; that is, calendar time in the statistical model will be the randomised crossover times irrespective of whether crossover actually occurred at the pre-specified time. If there are discrepancies between randomised and actual crossover times, a supporting analysis will use the actual time that crossover occurred. Results will be expressed as rates per 1000 occupied bed days with 95% confidence intervals.

Data from the transition months will be reported but not included in the final analysis. All data will be analysed using SAS software (SAS Institute Inc., Cary, NC, USA). For the primary analysis, a *P* value less than 0.05 will be considered statistically significant. A statistical analysis plan will describe in detail the pre-specified analysis for all efficacy and safety outcomes and be available in the public domain before database lock.

### Randomisation

The unit of randomisation for this trial is the RACF (not the resident). Once the 18 RACFs selected for the stepped-wedge trial have provided consent to participate, each RACF will be randomised to a transition date by a statistician (Fig. 3). RACFs will be advised of their scheduled start date for crossover to the AMS ENGAGEMENT bundle 3 months in advance. This allows time to make the necessary arrangements for the intervention (convene AMS team for the facility, undertake an AMS needs assessment using the NAT, select and localise elements of the AMS ENGAGEMENT bundle). Each facility will spend 1-month in transition before sequentially crossing over to the implementation phase. The maximum time a facility can be in the implementation will be 18 months and the minimum will be 1 month (Fig. 3).

### Allocation concealment and blinding

The randomisation schedule will be accessible only by the statistician. Once a RACF has been informed of their start date, blinding of the intervention to RACF staff and GPs servicing the facilities is not possible as the educational elements of the AMS ENGAGEMENT bundle directly targets this group of clinicians. The research team members working with RACFs during the observation period will also not find out a facility’s randomisation until 3 months prior to their crossover date. A statistician not involved in the day-to-day management of the trial will generate and maintain the random allocation sequence for RACFs to transition from control to intervention.

### Data collection, management and monitoring

De-identified data will be provided by participating RACFs. Based on data sourced from pharmacy dispensing reports, infection control registers and electronic records systems, RACFs will provide monthly reports on antibiotic dispensing (as a proxy measure for consumption); suspected infections; number and types of pathology tests conducted relating to suspected UTIs or *C. difficile* infections; and details of resident hospitalisations, including admission and discharge dates, reason for hospitalisation and any additional medications or diagnoses related to the hospitalisation. Baseline data will be collected on residents’ demographics, co-morbidities (supplied annually to the Commonwealth Department of Health (i.e. the Aged Care Funding Instrument)), number of regular medications and current antibiotics. Antibiotics include all systemic antibacterials (the Anatomical Therapeutic Chemical (ATC) level J01) and topical antimicrobial agents.

An appropriately trained and authorised delegate at each facility will undertake data collection and reporting. This delegate will de-identify resident data prior to uploading to The University of Queensland’s secure Research Data Management platform, where it will be transferred into a REDCap© database. Only authorised users at each facility will be able to upload data using a unique username and password. The trial database will be stored securely on The University of Queensland network. A central coordinating centre (the Australasian Kidney Trials Network in the Centre for Health Services Research at The University of Queensland) will perform research data management and data cleaning. Access to research data will only be granted to staff involved in the cleaning and analysis of research data. The central coordinating centre will conduct on-site and remote monitoring at each RACF, on a quarterly basis or more frequently if needed, to ensure the accuracy and consistency of data collection and data management. This will include the comparison of source and trial data. The study design is open label so unblinding will not occur.

### Economic evaluation

The primary economic analysis will be a cost analysis of the intervention period compared to the control period. The following costs will be factored into the cost analysis:

- Pathology and medication costs of RACF residents
- Available information on resident hospitalisations, including treatments administered
- Cost of providing nurse and general practitioner education
- Cost of telehealth service provision
- Staff time spent on other AMS activities, i.e. AMS team meetings and resident education.

Costs will be assigned to resource usage using Medicare Benefits Scheme and Pharmaceutical Benefits Scheme item numbers and cost weights for hospitalisations. A generalised linear mixed model using appropriate family and link for the underlying distribution will be used to compare groups and account for the clustering effect of the RACF.

To ascertain the impact of reduced antibiotic usage in RACFs, a cost-effectiveness model will be developed from the health service payer perspective. The surrogate outcome of reduced usage will be transformed to a final outcome of reduction in AMR infections in residents in order to assess the longer-term costs and outcomes over the lifetime of residents. The model will incorporate subsequent changes in second-line treatments, longer treatments and more diagnostics. This will be based on trial data supplemented with literature estimates and/or expert opinion where data is not available. Utility will be assigned according to population values for this cohort with decrements applied for adverse outcomes.

### Process evaluation of AMS ENGAGEMENT

A comprehensive process evaluation will be conducted to identify the primary needs of RACFs with respect to AMS and to explore the barriers and enablers of stewardship programme. A mixed-methods evaluation to understand the functioning of the AMS ENGAGEMENT interventions will use data collected during the stepped-wedge trial and qualitative data collected during and after the trial. The evaluation will examine implementation, mechanisms of impact and contextual factors using a RE-AIM framework (reach, effectiveness, adoption, implementation, maintenance) [45] (Supplementary Material).

Qualitative data collected specifically for the process evaluation will include transcripts of the first AMS meeting at each RACF, pre- and post-intervention staff surveys and post-completion interviews with AMS team members. The first AMS meeting will be a facilitated workshop with members of the AMS team to review the facility’s Needs Assessment. Meeting transcripts will be analysed using inductive thematic analysis to identify contextual factors at each facility, as well as barriers and enablers to AMS implementation. RACF staff will be invited to participate in a pre-implementation survey 3 months prior to their facility’s implementation date and within 3 months of trial completion. The surveys will elicit perspectives on organisational readiness, staff beliefs about antibiotic use and barriers to change, and the Conditions for Work Effectiveness Questionnaire II (CWEQII) [46] will be administered to measure change in structural empowerment of nursing staff. Semi-structured interviews will be conducted within 3 months of trial completion with a purposively selected sample of AMS team members to review barriers and enablers and to assess intervention maintenance. Individual consent will be provided by all workshop, survey and interview participants prior to their participation in qualitative data collection. Participant confidentiality will be protected through the use of pseudonyms for workshop and interview transcripts, and by not tracking individual survey responses.

### Challenges: retention of sites

Recruitment and retention of RACFs will be challenging in the midst of the COVID-19 pandemic. The following strategies will be adopted to optimise recruitment and retention:

- All components of the AMS ENGAGEMENT bundle will be designed for remote delivery through mediums such as print materials, videoconferencing, teleconferencing, webinars and online tutorials and videos (to mitigate the risk of COVID-related restrictions limiting participation).
- A representative from a RACF will be included on the Trial Steering Committee to ensure the protocol and interventions are designed and implemented with feasibility in mind.
- The research management team will be accessible to participating facilities during the planning and needs assessment to address questions and respond to any concerns.
- All interventions, including AMS team meetings, will be scheduled to suit the operational and care delivery needs of participating facilities.
- A dedicated member of the research team will maintain regular (at least monthly) contact with a liaison from each facility prior to and during the observation period to proactively identify and address any obstacles to participation.

The risk remains that facilities will discontinue their participation during the observation period for a number of reasons, including resourcing limitations, preference for early intervention of the AMS ENGAGEMENT bundle, COVID-19 impacts or changes in organisation or facility management. Additionally, aged care service providers with multiple RACFs enrolled in the trial may not want to wait to implement an AMS programme across all facilities if is it well received and/or proving effective. This risk may be mitigated by making the publicly accessible components of the AMS ENGAGEMENT bundle (e.g. guidelines) available to these facilities, but discouraging the sharing of facilitated interventions such as the education materials, telehealth webinars and nursing telephone support. The research team will also specifically request information on any AMS activities conducted at the facility at their regular monthly contact.

### Trial safety monitoring

Trial safety will be monitored by an internal safety reviewer, who will be an infectious disease expert associated with the trial (but not a member of the TSC). This decision is based on findings of a recent trial of an AMS programme in 12 US RACFs [18] and a systematic review which included studies that evaluated the impact of AMS on health outcomes and health care utilisation [34]. Both studies found no significant differences in all cause hospital admissions or mortality between RACFs who received AMS-related interventions and those that did not. The appointment of an internal safety reviewer is also consistent with the Australian *National Statement on Ethical Conduct in Human Research (2007)* (National Statement) which “permits monitoring arrangements to be commensurate to the risk, size and complexity of the trial. The nature and extent of participant safety monitoring should be based on the assessment of the risks of the trial intervention(s) relative to standard care and the extent of knowledge about the IMPs/IMDs being tested. The sponsor’s plans for safety monitoring should be documented and continually reviewed and adapted during the trial, as real time assessments of safety data are performed.”

The Central Coordinating Centre (UQ) will prepare reports for the safety reviewer on resident hospitalisation, infection and mortality rates based on RACF monthly data submissions. Reports will be supplied to the safety reviewer, who will request further information, where applicable, on any significant deviations (≥1SD) from the trend.

### Dissemination of findings

Dissemination of trial results will occur in accordance with the Trial Management Plan. Dissemination activities will include:

- Post-implementation workshops with participating RACFs and their governing bodies (where applicable)
- Academic papers, presentation at scientific conferences
- Final report with key findings and recommendations for a broader rollout of AMS activities in Australian RACFs.

No participating RACFs, their staff or residents will be identified during research dissemination activities.

### Protocol modifications

Any modifications to the protocol which may impact on the conduct of the trial, have potential benefit to the participant/s or may affect participant safety, including changes of trial objectives, trial design, trial population, sample size, trial procedures or significant administrative aspects, may require a formal amendment to the protocol. Such amendment will be agreed upon by the Trial Steering Committee and approved by the relevant ethics committee prior to implementation and will be communicated in writing and at quarterly meetings.

### Authorship policy

To qualify for authorship, a contributor should:

- Have made substantial contributions to the conception or design of the work; or the acquisition, analysis, or interpretation of data for the work; or the creation of new software used in the work; or have drafted the work and substantially revised it
- Have approved the submitted version (and any substantially modified version that involves the author’s contribution to the trial); AND
- Have agreed both to be personally accountable for the author’s own contributions and to ensure that questions related to the accuracy or integrity of any part of the work, even ones in which the author was not personally involved, are appropriately investigated, resolved, and the resolution documented in the literature.

All contributors who meet the first criterion will be given the opportunity to qualify for authorship.

## Discussion

RACFs have been identified as complex adaptive systems, with multiple influences on antimicrobial prescribing, including clinical staff, factors related to the facility and the resident and family wishes [13], all of which may play a role in over prescribing and the development of resistant organisms [14]. This is problematic given that RACF residents change care settings frequently, and resistant bacteria can then be transported between the facility and acute care settings increasing transmission of AMR [14]. A structured AMS model of care, incorporating targeted interventions to optimise antimicrobial use in the RACF setting, is urgently needed and will be delivered by our trial. The trial will aim to empower clinicians by providing a robust AMS programme to improve antibiotic-related health outcomes for aged care residents.

This trial will use a mixed methods approach to evaluate the impact of AMS stewardship in aged care and through our comprehensive process evaluation will enable findings to inform future research in this field for sustainable change.

### Trial status

The selection process for trial sites was undertaken in May and September 2021, and the selection process was completed in September 2021. The randomisation of sites commenced in September 2021 and is ongoing. Protocol Version: 1.2 - 9 July 2021

### Trial registration

The trial has been registered on the US National Library of Medicine Clinical Trials.gov (NCT04705259).

## Supplementary Information

**Additional file 1.** Appendix.

**Additional file 2: Supplementary material**. Process Evaluation Measures.

## Data Availability

For researchers outside the AMS ENGAGEMENT trial, de-identified data will be shared if there is approval by the Trial Steering Committee and the appropriate research proposal and ethical clearance are in place. This process will be in effect for a period of 2 to 5 years following publication of the main trial results. After 5 years, the data will be available in The University of Queensland’s data warehouse but without investigator support other than deposited metadata.

## Abbreviations

AMR: Antimicrobial Resistance
AMS: Antimicrobial Stewardship
ASB: Asymptomatic bacteriuria
DDDs: Defined daily doses
RACFs: Residential aged care facilities
RE-AIM: Reach, effectiveness, adoption, implementation, maintenance
UTIs: Urinary tract infections
