# Supplementary material for: A multimodal intervention to optimise antimicrobial use in residential aged care facilities (ENGAGEMENT): protocol for a stepped-wedge cluster randomised trial
Source: Trials. 2022 May 21;23:427. doi: 10.1186/s13063-022-06323-8 (PMC9123829; doi:10.1186/s13063-022-06323-8)
Supplement: Supplementary file 1 — Additional file 1. Appendix. [file 13063_2022_6323_MOESM1_ESM.zip › Supplementary materials_Appendix_AMSR2_2.docx]

| **Data** | **Information** |
| --- | --- |
| Primary registry and ID number | ClinicalTrial.gov identifier: NCT04705259 |
| Date of registration | November 2020 |
| Source of monetary support | National Health and Medical Research Council, Australia (Medical Research Futures Fund) |
| Secondary ID numbers (Unique protocol ID) | 2020002193 |
| Primary Sponsor | The University of Queensland (Centre for Health Services Research) |
| Contact for queries | Ms Alyssa Welch email: ams.engagementstudy@uq.edu.au |
| Public Title | A Multimodal Intervention to Optimise Antimicrobial Use in Residential Aged Care Facilities (ENGAGEMENT Study) |
| Scientific Title | A Multimodal Intervention to Optimise Antimicrobial Use in Residential Aged Care Facilities (ENGAGEMENT Study): Protocol for a Stepped Wedge Cluster Randomised Trial |
| Country of recruitment | Australian |
| Health Condition or Problem to be studied | Antibiotic consumption in aged care |
| Intervention | A bundle of multimodal interventions to optimise antibiotic use in aged care (including stewardship, education and implementation of guidelines) |
| Key inclusions and exclusions | Inclusions: Aged care facilities (RACFs) from licenced aged care organisations in south east Queensland, Australia with at least 50 residents  Exclusions: RACFs with less than 50 residents; RACFs unable to provide reports for baseline data |
| Study type | Stepped wedge randomised control study (interventional) |
| Target Sample Size | 18 aged care facilities with at least 50 residents |
| Start date | October 2021 |
| Primary Outcome | Defined Daily Doses of Antibiotics |
| Secondary Outcomes | Number of urine samples collected per 1000 resident bed days between the control vs intervention periods  Percent susceptibility of Enterobacteriaceae to ceftriaxone, ciprofloxacin, cephalexin and amoxicillin-clavulanate measured using antibiograms  All-cause on-year mortality rates of residential aged care facility (RACF) residents between the control vs intervention periods (per 1000 resident bed days and median rate across facilities)  Number of RACF residents admitted to hospital during the control vs intervention periods (per 1000 resident bed days and median rate across facilities) |

Trial Management

- 1. Organisational structure
     1. Trial Steering Committee (TSC)

The TSC will oversee the trial on behalf of the sponsor (the sponsor retains ultimate responsibility for the trial). The TSC will be responsible for trial design, finalisation of the protocol, delivery of the intervention, data collection, management, analysis, interpretation of data, writing the report, and submission of the final report as a manuscript for publication. The TSC will oversee the business and financial management of the trial. The Trial Steering Committee will retain sole decision-making regarding trial continuation and modifications to trial design and procedures while maintaining confidentiality of the accumulating data.

- - 1. Trial Management Committee

The Trial Management Committee provides support to the Trial Steering Committee in the execution of their responsibilities. Its focus is primarily on operational matters regarding the trial’s management and conduct. It provides regular reports to the Trial Steering Committee with information including site status, recruitment, retention, protocol compliance, data completeness and legal and regulatory issues.

- - 1. Clinical Advisory Committee

The Clinical Advisory Committee (CAC) will be led by Professor Len Gray. This Committee reports to the TSC, will be responsible for expert clinical oversight of the trial and will ensure the development of clinically appropriate interventions and resources.

- - 1. Central Coordinating Centre

The Australasian Kidney Trials Network (AKTN) at the Centre for Health Services Research, is the coordinating centre on behalf of the University of Queensland. Theywill be responsible for site set-up and close-out, training, monitoring, data management, statistics, distributions of funds to sites, and supporting the central operations including governance and committees.

- 1. Conflicts of interest

Investigators are asked to declare any conflicts of interest at the time of their appointment, and are responsible for notifying AKTN of any conflicts that arise during the trial. Any conflicts will then be mitigated or eliminated accordingly to ensure the trial’s integrity. The Trial Steering Committee, aided by the Trial Management Committee, will have oversight of this.

## Trial Steering Committee

| **Member** | **Institution** |
| --- | --- |
| Professor Len Gray | CHSR, UQ |
| Professor David Paterson | UQCCR, UQ |
| Professor Carmel Hawley | CHSR, UQ |
| Professor Ruth Hubbard | PAH Southside Clinical Unit, UQ |
| Associate Professor Christopher Freeman | School of Pharmacy, UQ |
| Ms Elaine Pascoe | CHSR, UQ |
| Doctor Ellen Burkett | Department of Emergency Medicine, PAH |
| Associate Professor Tracy Comans | CHSR, UQ |
| Dr Nazanin Falconer | CHSR, UQ, |
| Ms Alyssa Welch | CHSR, UQ |

## Trial Management Committee

| **Member** | **Institution** | **Role** |
| --- | --- | --- |
| Dr Nazanin Falconer | The University of Queensland | Research Fellow |
| Dr Leila Shafiee Hanjani | The University of Queensland | Research Fellow |
| Professor Len Gray | The University of Queensland | Clinical Investigator and Lead |
| Ms Alyssa Welch | The University of Queensland | Clinical Project Manager |
| Mr Ronvy Arimbuparambil | The University of Queensland | Database Administrator |
| Dr Leila Shafiee Hanjani | The University of Queensland | Research Fellow |
| Ms Elaine Pascoe | The University of Queensland | Statistician |
| Ms Donna Reidlinger | The University of Queensland | Clinical Operations Manager |

1. Data Management
   1. Data transmission and storage

De-identified resident data will be uploaded to a secure UQ drive, such as Research Data Manager (RDM), by an appropriately trained and authorised delegate (reporting officer) at each facility (or at the parent organisation level if relevant). Only authorised users at each facility/organisation will be able to upload data using a unique username and password. The trial database will be stored securely on The University of Queensland network. Research data management will be performed by the Central Coordinating Centre (the AKTN) and data cleaning will be performed by a research assistant independent of the research team. Access to research data will be granted only to staff involved in the cleaning and analysis of research data. To ensure the accuracy and consistency of data collection and data management, a data management expert will visit and monitor sites.

Investigators will be required to maintain all trial documentation, including consent documents, ethics committee approvals and correspondence, for a period of 7 years after the closure of the trial. All other research data will be stored for 15 years from the end of the trial period and then destroyed.
