# Supplementary material for: A multimodal intervention to optimise antimicrobial use in residential aged care facilities (ENGAGEMENT): protocol for a stepped-wedge cluster randomised trial
Source: Trials. 2022 May 21;23:427. doi: 10.1186/s13063-022-06323-8 (PMC9123829; doi:10.1186/s13063-022-06323-8)
Supplement: Supplementary file 2 — Additional file 2: Supplementary material. Process Evaluation Measures. [file 13063_2022_6323_MOESM2_ESM.docx]

Supplementary materials: Process Evaluation Measures

| **RE-AIM** | **Process outcome** | **Data source** |
| --- | --- | --- |
| **Reach** | Number and characteristics of nursing staff, and affiliated GPs and pharmacists who participate in each component of the AMS ENGAGEMENT intervention bundle. | Trial records |
|  | Clinician willingness to adopt changes in practice to improve use of antibiotics | Pre-implementation staff survey |
|  | Challenges and enablers: trust in information and training, trust in service provider, practicality of changes to current circumstances | Pre-implementation staff survey |
| **Effectiveness** | Clinical outcomes and RACF performance – individual and aggregate | Trial final paper/s |
|  | Benefits and harms, including unintended benefits and harms | Trial records, safety officer reports, post-implementation interviews |
|  | Challenges and enablers: factors not directly measured that may have impacted changes in DDD | AMS meeting minutes, post-implementation interviews |
| **Adoption** | Influence of facility selection on trial outcomes (includes representativeness of participating facilities compared to aged care sector) | Annual and Commonwealth reports on Aged Care |
|  | Challenges and enablers: considerations of RACFs when deciding their suitability / capacity for trial participation | Trial manager notes, emails, post-completion interviews, AMS meeting minutes |
| **Implementation** | Integration of AMS changes into standard resident care | Monthly observations, AMS meetings, post-implementation interviews |
|  | Delivery of ENGAGEMENT Bundle components (including fidelity)  Measures include: Number of nurses at each facility; number who successfully pass the online quiz at the end of the online education modules; Number of GPs affiliated with each facility; Number of GPs who complete the academic detailing; Number of AMS team members; Number of AMS team meetings | AMS meeting minutes, trial database records, monthly observations |
|  | Suitability of trial protocol to RACF environment | NAT results, protocol variations, protocol amendments, trial database records, post-implementation staff survey |
|  | Challenges and enablers: practicality of implementing AMS  In particular, influence of external policy environment on trial fidelity (e.g. Royal Commission into Aged Care) | Observations, AMS workshop, AMS meeting minutes, post-completion interviews |
|  | Resource use | Trial database records, health economic evaluation, trial management records |
| **Maintenance** | Sustainability: irrespective of trial outcomes whether RACFs would consider adopting AMS changes and any adaptations they would make / have made. | Post-completion interviews, observations, AMS meeting minutes |

Key: AMS - antimicrobial stewardship; DDD - defined daily doses; GP - general practitioner; NAT - needs assessment toolkit; RACFs -Residential Aged Care Facilities
